# Community dynamics of nematodes after Larsen ice‐shelf collapse in the eastern Antarctic Peninsula

**DOI:** 10.1002/ece3.1869

**Published:** 2015-12-29

**Authors:** Freija Hauquier, Laura Ballesteros‐Redondo, Julian Gutt, Ann Vanreusel

**Affiliations:** ^1^ Marine Biology Research GroupGhent UniversityKrijgslaan 281B‐9000GhentBelgium; ^2^ Alfred Wegener InstituteHelmholtz Centre for Polar and Marine ResearchAm Handelshafen 12D‐27570BremerhavenGermany

**Keywords:** Biodiversity, colonization, community dynamics, *Halomonhystera*, ice‐shelf collapse, Larsen B, marine free‐living Nematoda, *Microlaimus*

## Abstract

Free‐living marine nematode communities of the Larsen B embayment at the eastern Antarctic Peninsula were investigated to provide insights on their response and colonization rate after large‐scale ice‐shelf collapse. This study compares published data on the post‐collapse situation from 2007 with new material from 2011, focusing on two locations in the embayment that showed highly divergent communities in 2007 and that are characterized by a difference in timing of ice‐shelf breakup. Data from 2007 exposed a more diverse community at outer station B.South, dominated by the genus *Microlaimus*. On the contrary, station B.West in the inner part of Larsen B was poor in both numbers of individuals and genera, with dominance of a single *Halomonhystera* species. Re‐assessment of the situation in 2011 showed that communities at both stations diverged even more, due to a drastic increase in *Halomonhystera* at B.West compared to relatively little change at B.South. On a broader geographical scale, it seems that B.South gradually starts resembling other Antarctic shelf communities, although the absence of the genus *Sabatieria* and the high abundance of *Microlaimus* still set it apart nine years after the main Larsen B collapse. In contrast, thriving of *Halomonhystera* at B.West further separates its community from other Antarctic shelf areas.

## Introduction

The Antarctic Peninsula is one of the most affected areas worldwide by rapid regional warming (Vaughan et al. 2003), and this has led, among other things, to large‐scale ice‐shelf destabilization and disintegration. The Larsen area east of the Peninsula is one of the regions where ice‐shelf collapse is evident: in 1995, the Larsen A ice shelf (LIS‐A) disintegrated almost completely, and in February–March 2002, the Larsen B ice shelf (LIS‐B) lost with roughly 3250 km² the largest proportion of its surface after a decade of several smaller disintegration events and millennia of stability (Rack and Rott 2004; Domack et al. 2005; Rebesco et al. 2014). The sudden collapse of LIS‐B was mainly attributable to surface processes, rather than basal melting in response to oceanic warming (Gilbert and Domack 2003; Vaughan et al. 2003; Rack and Rott 2004; Scambos et al. 2004; Rebesco et al. 2014). Prior to the actual breakup, there had been an exceptionally warm summer and the surface net mass balance of the ice shelf had been decreasing for several years (Rack and Rott 2004). This eventually led to ice thinning and the formation of meltwater ponds and crevasses at the surface, further enhancing rapid disintegration (Gilbert and Domack 2003; Rack and Rott 2004). Currently, the remnant LIS‐B (and its tributary glaciers; Rott et al. 2011; Berthier et al. 2012) continues to decrease, evidenced by an additional loss of 50% of the initial collapsed area over the period 2002–2009 (Shuman et al. 2011).

Sudden ice‐shelf collapse results in profound changes for associated marine benthic ecosystems. In areas like Larsen (e.g., the western Antarctic Peninsula; Moline et al. 2004; Clarke et al. 2007), loss of permanent shelf ice enables phytoplankton to bloom in areas previously ice‐locked for several millennia (Bertolin and Schloss 2009; Barnes and Clarke 2011). Furthermore, ice algae released upon seasonal ice melt may provide a valuable additional food source, especially in seasonally opened polynyas nearby the continent (Cape et al. 2014). Together, both processes enhance direct fresh food supply to seafloor‐dwelling organisms, triggering colonization of previously ice‐covered habitats from nearby sources. On the downside, sudden ice‐shelf decay increases the risk of iceberg scouring as large icebergs break off and ground in areas further offshore (Gutt et al. 1996; Lee et al. 2001).

Despite all efforts in the study of benthic response to climate‐induced events such as ice‐shelf collapse and iceberg scouring, considerable uncertainty remains on how biodiversity is affected by, and what the resultant ecological responses are of these processes. To gain long‐term information, several benthic faunal components of Larsen B were sampled during two expeditions onboard the German icebreaking *RV Polarstern* in austral summer of 2007 (ANT‐XXIII/8) and 2011 (ANT‐XXVII/3). Meiobenthos (32–1000 *μ*m) of the first expedition was assessed by Raes et al. (2010) and Hauquier et al. (2011), focusing on the numerically most important Nematoda. Already then, 5 years after the main LIS‐B collapse, significant differences were observed between Larsen stations for all faunal groups, driven by different response rates to the change from an oligotrophic sub‐ice shelf to a more productive ecosystem (Gutt et al. 2011). Based on faunal abundance and diversity, stations B.South located at the original ice‐shelf edge and B.West in the middle of the embayment contrasted most. For Nematoda, this observation was explained by a combination of the duration of the ice‐free period and the connection with precollapse open Weddell Sea conditions (Raes et al. 2010). The main objective of expedition ANT‐XXVII/3 in 2011 was to revisit 2007 locations and look at benthic ecosystem recovery and dynamics. This study re‐analyses 2007 data for stations B.South and B.West and compares them with new (i.e., 2011) nematode community data to resolve nematode community response to ice‐shelf collapse on a longer time scale. Given continued increase in vertical food supply and exchange with the open Weddell Sea, we hypothesize that:

1. Abundance and diversity at B.West will increase and nematode communities at both locations will converge in terms of numbers, diversity, and generic composition,
2. Communities within Larsen B will increasingly resemble other Antarctic shelf areas of similar water depth that do not necessarily share the same history of permanent ice shelter.

## Material and Methods

### Sampling area and strategy

**Figure 1:**
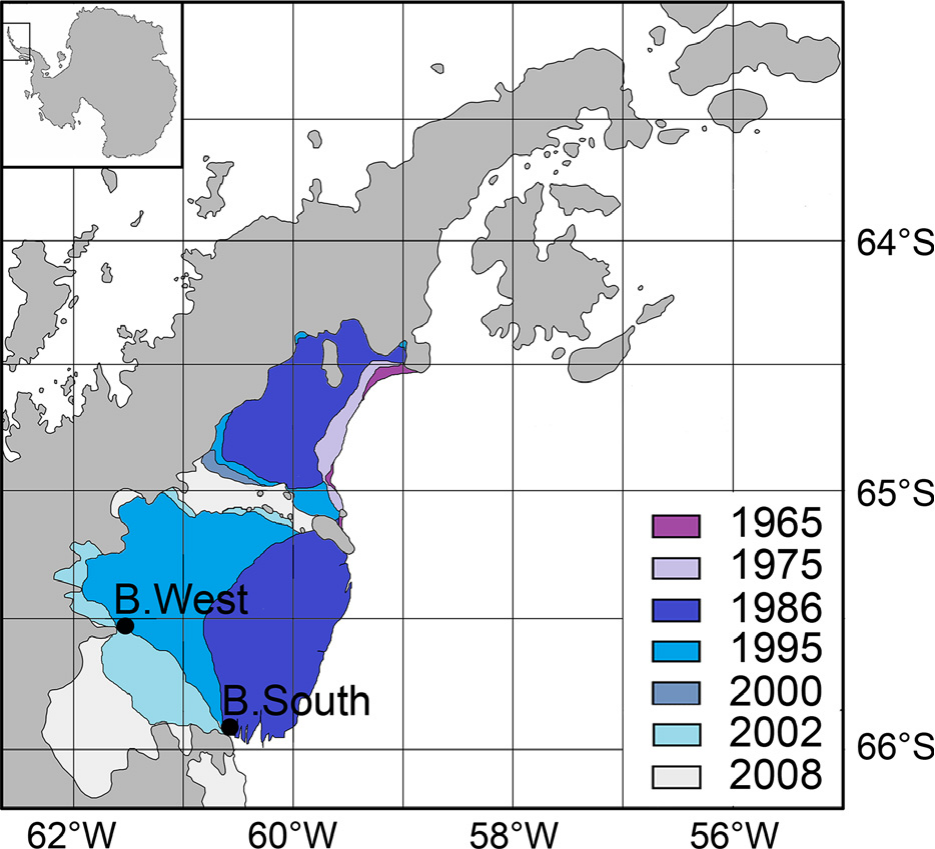
Sampling locations B.West and B.South and the evolution of the ice‐shelf extent over selected years.

Stations B.South and B.West of *Polarstern* expedition ANT‐XXIII/8 (January 2007) were re‐sampled during ANT‐XXVII/3 (March 2011) using five random replicate multicorer deployments (MUC, inner diameter 57 mm; Barnett et al. 1984) per location, allowing for equivalent and comparable sample coverage (Table 1, Fig. 1). B.South was always located at the border of the original ice shelf in open connection to the Weddell Sea (hence referred to as “outer” station), whereas B.West (inner station) experienced permanent ice cover until after the 2002 collapse (evolution of ice‐shelf extent is depicted in Fig. 1; see also Raes et al. 2010).

**Table 1:** Geographic position and depth of Larsen B.South and B.West replicates, both for ANT‐XXIII/8 (2007) and ANT‐XXVII/3 (2011)

Station	2007				2011			
	Replicate	Latitude	Longitude	Depth (m)	Replicate	Latitude	Longitude	Depth (m)
B.South	700‐8	65° 54.98′ S	60° 20.54′ W	422	246‐3	65° 54.95′ S	60° 20.43′ W	424
	700‐9	65° 54.95′ S	60° 20.88′ W	417	246‐4	65° 54.95′ S	60° 21.49′ W	395
	702‐4	65° 55.12′ S	60° 19.96′ W	427	246‐5	65° 54.99′ S	60° 20.70′ W	419
	702‐7	65° 54.49′ S	60° 21.37′ W	405	247‐3	65° 55.12′ S	60° 19.83′ W	428
	702‐8	65° 54.95′ S	60° 20.95′ W	410	247‐4	65° 55.15′ S	60° 20.01′ W	425
B.West	710‐2	65° 33.03′ S	61° 36.98′ W	277	233‐4	65° 32.99′ S	61° 36.94′ W	277
	710‐3	65° 33.04′ S	61° 37.18′ W	281	233‐5	65° 32.97′ S	61° 36.94′ W	278
	710‐7	65° 33.03′ S	61° 37.01′ W	275	235‐4	65° 32.96′ S	61° 36.88′ W	276
	710‐8	65° 33.03′ S	61° 37.00′ W	283	235‐5	65° 33.01′ S	61° 36.96′ W	280
	710‐9	65° 33.07′ S	61° 37.06′ W	288	235‐6	65° 33.01′ S	61° 37.00′ W	279

The top 0–5 cm of one core per replicate deployment was sliced at a 1‐cm resolution and preserved in 4–8% formalin for meiofauna analysis. Table 1 gives the geographic position and depth of the 2011 and – for ease of comparison – the 2007 samples. Meiofauna was extracted from the sediment using 1 mm and 32 *μ*m sieves and density gradient centrifugation with Ludox (specific density 1.18 g cm^−^³; Heip et al. 1985; Vincx 1996), fixed in 4% formalin, and dyed with Rose Bengal (0.5 g L^−1^). All meiofauna was counted and identified at higher taxon level using a stereomicroscope and the guide of Higgins and Thiel (1988).

From each layer, 150 nematodes were randomly picked (or all when the number of nematodes <150), transferred to anhydrous glycerol (Seinhorst 1959), and mounted on slides. Genus‐level identification (using a Leica DMLS compound microscope, 1000× magnification) was based on the pictorial key of Warwick et al. (1998) and the NeMYS database (Vanaverbeke et al. 2014).

As for 2007, samples for faunal analysis were complemented with an additional sample set for the measurement of environmental variables. These were analyzed at a coarser vertical resolution, 0–3 cm and 3–5 cm. Sediment grain size distribution was determined by laser diffraction (Malvern Mastersizer 2000, size range 0.02–2000 *μ*m) and classified following Wentworth (1922). Granulometric variables considered in this study were median grain size, silt (<63 *μ*m) and sand (>63 *μ*m) percentage. Pigments were extracted from lyophilized sediments by adding 10 mL 90% acetone, and chlorophyll *a* (chl*a*;* μ*g g^−1^) was measured with a fluorescence detector after HPLC (high‐performance liquid chromatography) separation. Additionally, total organic carbon (TOC) and nitrogen (TN) fractions were measured on 2011 freeze‐dried samples using a Flash 2000 organic elemental analyzer (protocol available through Interscience B.V., Breda, The Netherlands). Their ratio was calculated and multiplied by 14:12 to account for the difference in molar mass (C:N_molar_). Finally, sediment total organic matter (TOM) was determined after combustion at 550°C.

### Statistical analyses

Nematode abundance and community composition in 2011 were analyzed both separately and in conjunction with 2007 data. Analyses were executed in PRIMER v6 (Clarke and Gorley 2006) with the PERMANOVA+ add‐on (Anderson et al. 2008), unless mentioned otherwise. Nematode assembly data were standardized to individuals per 10 cm^2^ (ind.10 cm^−2^) and square‐root transformed to limit influence of dominant genera.

Differences in communities between areas and sediment depth layers in 2011 were assessed using a PERMANOVA (permutational ANOVA) design with two fixed factors (area, layer; Bray–Curtis similarity of genus ind. 10 cm^−2^; 9999 permutations) and visualized using PCO (principal coordinate analysis). SIMPER (similarity of percentages) identified which genera were responsible for (dis)similarities between samples. Community data were then summed for 0–3 and 3–5 cm depth for each replicate preceding correlation with environmental variables (as these were measured at a rougher scale) and averaged for both areas. TOM was log‐transformed to reduce right‐skewness, and sand content was omitted from the analysis owing to its high correlation (*r* > 0.9) with silt. All environmental variables were normalized. BEST analysis quantified the correlation between environmental setting and nematode assemblages.

Comparison of 2011 and 2007 data was made by PERMANOVA. Univariate analysis of nematode densities used a two‐factor design (area, year = fixed; Euclidean distances of nematode ind.10 cm^−2^, 9999 permutations), multivariate nematode composition data a three‐factor design (area, year, sediment depth = fixed; Bray–Curtis similarity of genus ind.10 cm^−2^, 9999 permutations). Pairwise tests were performed between all pairs of levels for significant factors. When the number of unique permutations exceeded 100, true permutational *P*‐values were reliable. When this number was below 100, Monte Carlo *P*‐values were interpreted. Results were accompanied by a PCO graph, combined with CLUSTER results, to gain visual insight in the data cloud.

Diversity indices (*N*_0_ = number of genera; *H*' = Shannon index (log*e*); *EG(200) = *expected number of genera in a sample of 200 individuals; Hill's *N*_1_) and evenness (Hill's *N*_inf_; *J' *= Pielou's evenness) were calculated in accordance with Raes et al. (2010). The rarefaction index *EG(n)* was based on 200 as the lowest number of identified specimens in one of the replicates was 215 (Clarke and Gorley 2006). After assumption testing in R (R Core Team, 2013), several indices did not fulfill requirements for two‐way ANOVA; hence, differences in diversity between areas and years were assessed using PERMANOVA (design identical to that for abundance data).

Finally, the 2007 and 2011 Larsen data were included in a larger dataset on (sub)‐Antarctic nematode shelf assemblages (0–1000 m) to examine relationships within a broader geographical context (Table 7). Data were grouped over larger geographical scales to simplify analysis. Groupings were chosen arbitrarily, disregarding geographical coordinates, and should not be interpreted as true biogeographical provinces. One‐way ANOSIM (analysis of similarity) assessed differences between areas, which were visualized with nonmetric MDS (multidimensional scaling).

## Results

### Nematode abundance and vertical distribution

**Figure 2:**
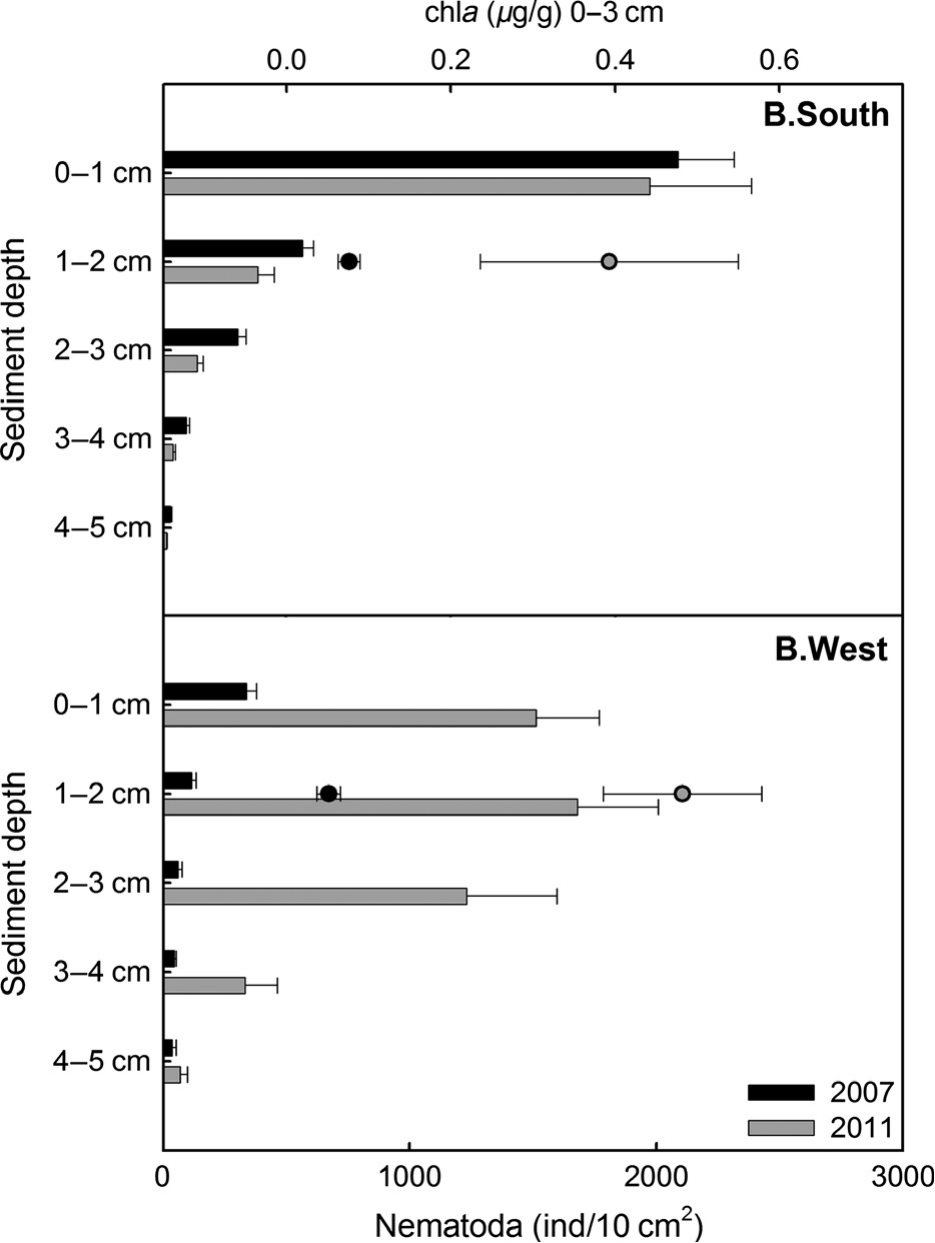
Average vertical nematode abundance (bars) and surface (0–3 cm) chlorophyll *a* values (dots) at stations B.West and B.South in 2007 (black) and 2011 (gray). Error bars indicate standard error (standard deviation/√number of replicates).

In all 2011 samples, regardless of their location or sediment depth, nematodes formed the most abundant meiofaunal taxon (relative contribution 93–95%). Whereas nematode total densities (i.e., summed over 0–5 cm) in both areas differed a lot in 2007, they were comparable in 2011 (and no longer significantly different; Tables 2 and 3: pairwise tests for factor area). This is the result of a clear increase in total nematode densities at B.West, and a slight (but insignificant) decrease at B.South (Tables 2 and 3: pairwise tests for factor year). Also nematode vertical distribution differed between stations and years (Fig. 2). Vertical profiles showed steep declines with depth in both years for B.South. Profiles were less steep at B.West, especially in 2011 when nematode density peaked at 1–2 cm and remained relatively high down to 4 cm depth.

**Table 2:** Overview of total nematode density (ind.10 cm^−2^), diversity (*N*_0_, *EG(200)*,* H'*,* N*_1_), and evenness (*N*_inf_, *J'*), averaged for five replicates per area × year combination. Values in brackets represent standard deviation

	Density (ind. 10 cm^−2^)	*N*_0_	*EG(200)*	*H'*	*N*_1_	*N*_inf_	*J'*
2011							
B.South	2547.81 (472.38)	35.60 (4.56)	24.13 (3.44)	2.29 (0.32)	10.23 (3.10)	2.38 (0.51)	0.64 (0.07)
B.West	4832.24 (1038.26)	10.80 (2.39)	6.24 (1.35)	0.40 (0.16)	1.51 (0.26)	1.09 (0.06)	0.17 (0.05)
2007							
B.South	3075.94 (235.34)	29.40 (1.52)	24.63 (0.82)	2.53 (0.08)	12.57 (1.01)	3.16 (0.41)	0.75 (0.02)
B.West	604.71 (63.03)	20.80 (4.97)	16.90 (4.36)	1.57 (0.32)	4.99 (1.58)	1.89 (0.32)	0.52 (0.06)

**Table 3:** Two‐factor PERMANOVA main and pairwise test results for univariate parameters

	Density		*N*_0_		*EG(200)*		*H'*	
	Pseudo‐F/t	*P* (perms)	Pseudo‐F/t	*P* (perms)	Pseudo‐F/t	*P* (perms)	Pseudo‐F/t	*P* (perms)
Main test								
Area	0.027	ns (>9000)	104.26	*** (>9000)	98.279	*** (>9000)	174.52	*** (>9000)
Year	10.041	** (>9000)	1.3495	ns (>9000)	18.703	*** (>9000)	42.733	*** (>9000)
Area × Year	16.6	*** (>9000)	24.527	*** (>9000)	15.459	** (<9000)	18.331	** (>9000)
Pairwise test								
Area								
2007	10.144	*** (126)	3.701	** (21)	3.895	** (126)	6.603	** (126)
2011	1.999	† (123)	10.772	*** (40)	10.826	** (126)	11.871	** (126)
Year								
B.South	1.001	ns (123)	2.885	* (13)	0.321	ns (126)	1.653	ns (126)
B.West	4.061	** (126)	4.056	** (26)	5.221	** (126)	7.398	** (126)

	*N*_1_		*N*_inf_		*J'*	
	Pseudo‐F/t	*P* (perms)	Pseudo‐F/t	*P* (perms)	Pseudo‐F/t	*P* (perms)
Main test						
Area	100.66	*** (>9000)	61.446	*** (>9000)	196.12	*** (>9000)
Year	12.834	** (>9000)	23.622	*** (>9000)	83.039	*** (>9000)
Area × Year	0.485	ns (>9000)	0.0025	ns (>9000)	22.775	*** (>9000)
Pairwise test						
Area						
2007	–	–	–	–	7.444	** (126)
2011	–	–	–	–	11.967	** (126)
Year						
B.South	–	–	–	–	3.278	** (126)
B.West	–	–	–	–	9.263	** (126)

Significance codes: ***<0.001; **<0.01; *<0.05; ^†^<0.1; ns = nonsignificant.

Pseudo‐F/t = effect size; *P* (perms) = permutational (perms > 100) or Monte Carlo (perms < 100) *P*‐value. Numbers in brackets represent the number of unique permutations.

### Nematode community composition

**Figure 3:**
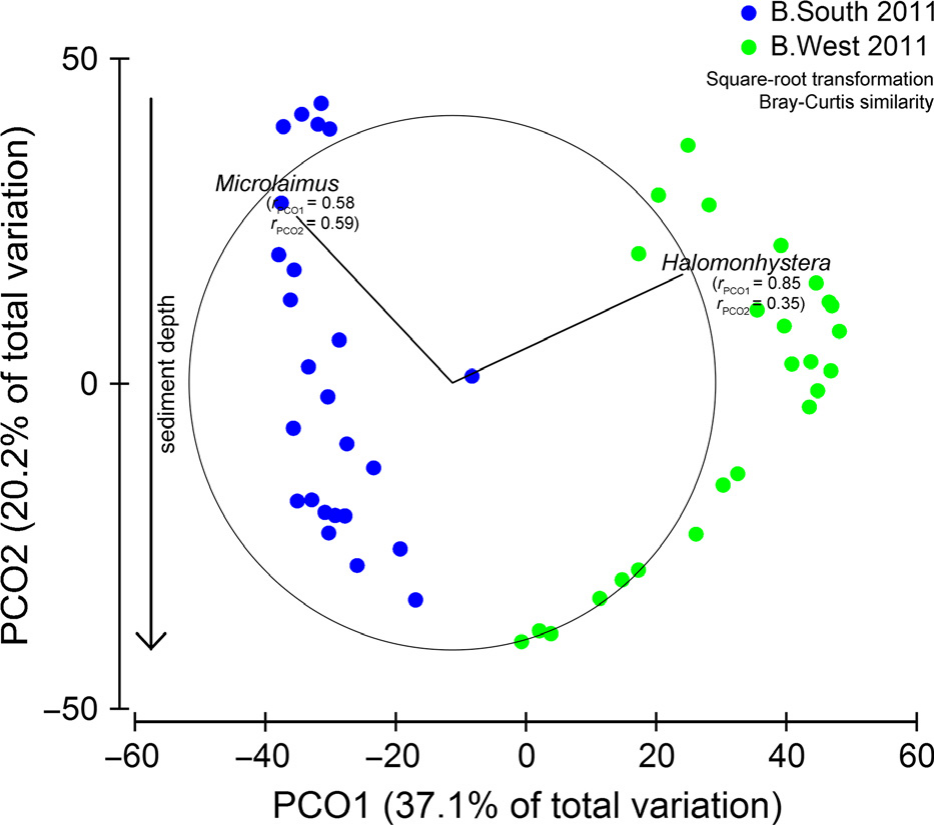
PCO of square‐root transformed nematode ind. 10 cm^−2^ in 2011. Vector overlays are genera with correlation >0.77 with the resulting plot. For each genus, its correlation with both PCO axes is indicated.

Nematode community composition in 2011 differed significantly between stations and cm‐layers (two‐factor PERMANOVA, significant interaction “area × layer”; results not shown) with largest differences between surface layers, gradually declining when moving deeper into the sediment (all pairwise *P* < 0.05). This is visible in the PCO plot for both stations in 2011 (Fig. 3). The first PCO axis (37.1% variation) divides samples according to their location, while the second axis (20.2% variation) is related to sediment depth. The dominant genera were *Microlaimus* at B.South and (a single species of) *Halomonhystera* at B.West (Table 4), which together explained almost half of the dissimilarity between both stations (average dissimilarity = 85.58%; contribution *Halomonhystera* + *Microlaimus* = 46.92%; SIMPER).

**Table 4:** Overview of the dominant genera at each station in 2007 and in 2011 (only genera with relative abundance >1% are included)

B.South				B.West			
2007		2011		2007		2011	
Genus	%	Genus	%	Genus	%	Genus	%
*Microlaimus*	32.20	*Microlaimus*	23.65	*Halomonhystera*	57.88	*Halomonhystera*	94.02
*Metadesmolaimus*	10.98	*Monhystrella*	14.98	*Thalassomonhystera*	21.00		
*Paracanthonchus*	9.90	*Halomonhystera*	14.89	*Theristus*	3.83		
*Halomonhystera*	9.09	*Chromadorita*	10.36	*Acantholaimus*	3.17		
*Monhystrella*	4.23	*Leptolaimus*	6.65	*Daptonema*	2.28		
*Neochromadora*	3.11	*Dichromadora*	5.19	*Monhystrella*	1.97		
*Prochromadorella*	3.09	*Acantholaimus*	4.32	*Desmodorella*	1.83		
*Araeolaimus*	3.07	*Thalassomonhystera*	2.62	*Halalaimus*	1.19		
*Acantholaimus*	2.78	*Daptonema*	2.45				
*Thalassomonhystera*	2.35	*Halichoanolaimus*	1.83				
*Theristus*	2.00	*Syringolaimus*	1.36				
*Leptolaimus*	1.87	*Cervonema*	1.23				
*Elzalia*	1.42	*Amphimonhystrella*	1.15				
*Daptonema*	1.33						
*Desmodorella*	1.30						
*Halichoanolaimus*	1.27						
*Dichromadora*	1.18						
*Desmodora*	1.10						

**Figure 4:**
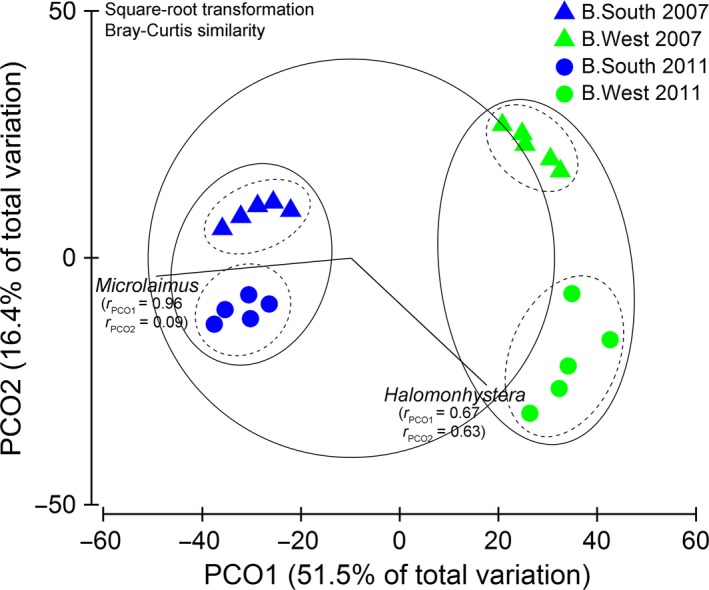
PCO and CLUSTER analysis. Plot based on square‐root transformed total ind. 10 cm^−2^ for each replicate of both stations and years (triangles = 2007, circles = 2011). Contours indicate 40 (full) and 60% (dashed) similarity levels as calculated by CLUSTER. Vectors show overlays of *Microlaimus* and *Halomonhystera*, with their respective correlations with PCO axes.

These two genera were also responsible for the clear separation between stations in terms of years (Fig. 4), as they remained most abundant at their respective area (Table 4). As a result, genus composition at B.South was relatively similar in 2007 and 2011, apart from some smaller differences (e.g., no *Metadesmolaimus* in 2011, Table 4). Also diversity and evenness remained fairly similar over the years (Table 2), with few significant differences (only *N*_1_ and *J'*; Table 3: pairwise tests for factor year). On the contrary, diversity and evenness at B.West were even lower than in 2007 (and differences were always significant; Table 3), due to a profound increase in *Halomonhystera*, mainly in the upper two centimeters of sediment (Table 4; Fig. 2). Hence, as was the case in 2007, genus diversity and evenness remained highest at B.South.

**Figure 5:**
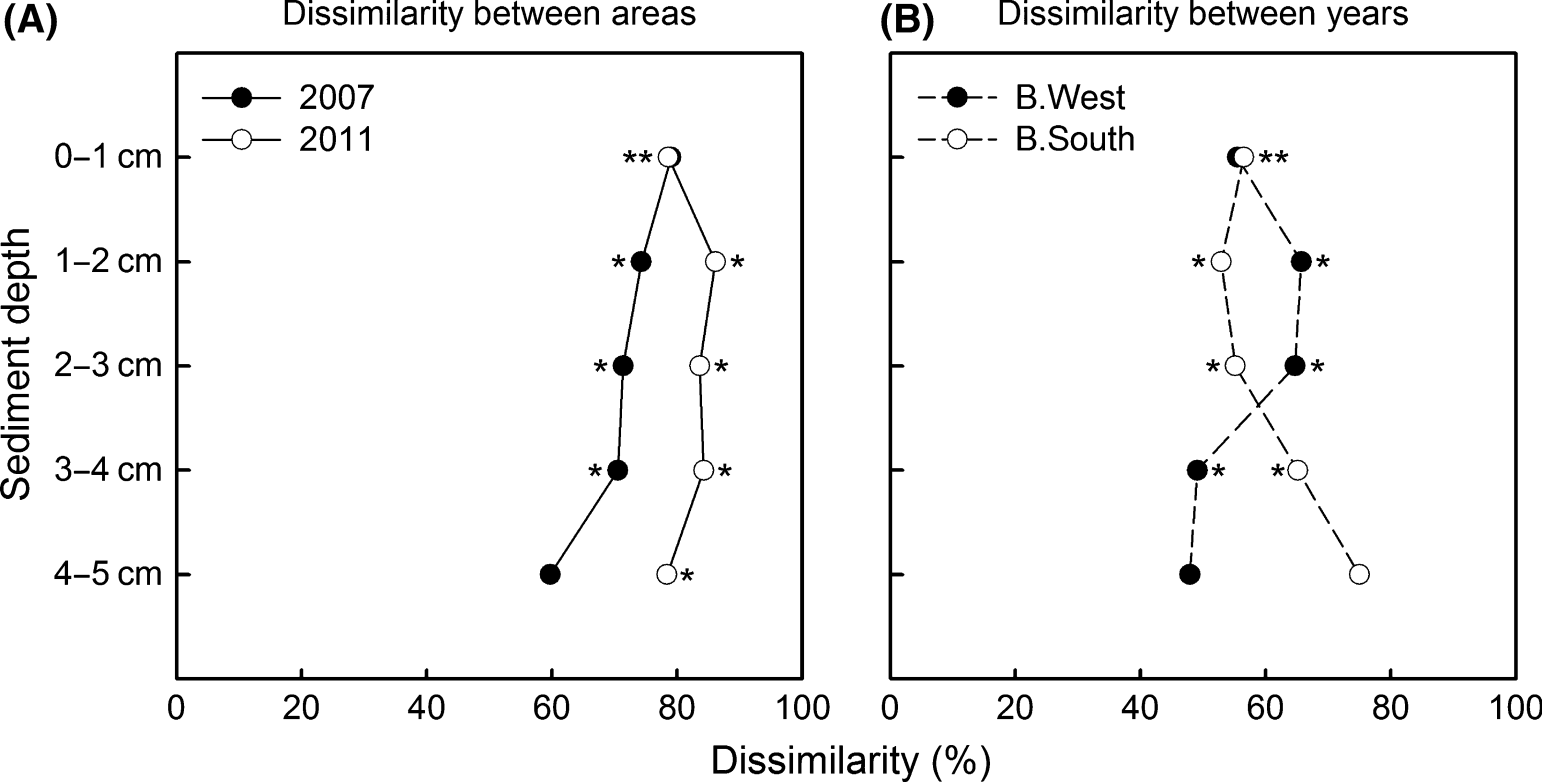
Visualization of PERMANOVA three‐way interactions. (A) Dissimilarities (%) between stations for each layer in 2007 (black) and 2011 (white). (B) Dissimilarities (%) between years at B.West (black) and B.South (white). Asterisks indicate significant differences (pairwise *P*‐values <0.05).

Three‐factor PERMANOVA, including sediment depth, showed that the differences in nematode assemblages between areas and years further depended on depth in the sediment (significant three‐way interaction, *P* < 0.05; Table 5). Communities of both areas differed mostly in surface layers and became more similar with depth (Fig. 5A: pairwise differences between stations for all levels of factors “year” and “layer”). This trend was more obvious in 2007 since communities in 2011 were more distinct in almost all depth layers. Alternatively, communities of both years became more similar at B.West with increasing depth, while the opposite occurred at B.South (Fig. 5B: pairwise dissimilarities across all levels of factors “area” and “layer”). This means that nematode assemblages in deeper layers of B.South diverged over the years, while they increasingly resembled each other at B.West (due to the large *Halomonhystera* contribution in all sediment layers in 2011).

**Table 5:** Three‐factor PERMANOVA main test results for nematode community data (ind. 10 cm^−2^)

Source	df	Pseudo‐F	*P* (perm)	Perms
Area	1	57.055	***	9936
Year	1	15.321	***	9930
Layer	4	15.404	***	9877
Area × Year	1	13.625	***	9917
Area × Layer	4	6.594	***	9868
Year × Layer	4	2.543	***	9845
Area × Year × Layer	4	2.507	***	9855
Res	80			
Total	99			

Df, degrees of freedom; Pseudo‐F, effect size; *P* (perm), permutational *P*‐value; Perms, number of unique permutations.

Significance codes: ***<0.001.

### Environmental setting

Averaged environmental variables for each layer in both stations and years (where available) are given in Table 6 (data grouped over 0–3 and 3–5 cm for 2007; *n*(2007) = 5; *n*(2011) = 2). Silt was the dominant grain size for all layers at both locations. B.South had a slightly higher sand content in 2007, but only for the upper centimeters. The biggest difference was a significant increase in chl*a* from 2007 to 2011, for both B.South and B.West (Fig. 2). Chl*a* content was higher in surface layers (0–3 cm) than deeper down (3–5 cm). Chl*a* values in 2011 alone did not differ much between stations, only between sediment layers. B.South samples had about twice as much TOC and TOM than B.West, leading to a higher C:N_molar_ as well (2011 only). BEST routine attributed 64% of 2011 nematode community variation to a combination of chl*a* and TOC.

**Table 6:** Average (standard deviation) values of environmental variables for 2007 and 2011 for each station, both divided in two layers, 0–3 cm and 3–5 cm. *n* = 5 for 2007 and *n* = 2 for 2011 samples

	MGS (*μ*m)	Silt% (<63 *μ*m)	Sand% (>63 *μ*m)	chl*a* (*μ*g g^−1^)	TOM (wt%)	TOC (wt%)	TN (wt%)	C:N molar
2011								
B.South 0–3 cm	19.50 (2.09)	96.09 (0.71)	3.91 (0.71)	0.39 (0.22)	0.08 (0.06)	0.58 (0.02)	0.06 (0.00)	11.57 (0.09)
B.South 3–5 cm	11.19 (6.52)	98.64 (1.92)	1.36 (1.92)	0.03 (0.00)	0.05 (0.01)	0.57 (0.01)	0.06 (0.00)	11.60 (0.17)
B.West 0–3 cm	18.15 (9.95)	99.70 (0.27)	0.30 (0.27)	0.48 (0.14)	0.03 (0.00)	0.25 (0.03)	0.05 (0.02)	7.05 (2.56)
B.West 3–5 cm	8.74 (0.40)	99.85 (0.03)	0.15 (0.03)	0.06 (0.04)	0.02 (0.01)	0.21 (0.03)	0.10 (0.04)	5.16 (2.84)
2007								
B.South 0–3 cm	34.34 (14.69)	90.56 (4.76)	9.44 (4.76)	0.08 (0.03)	–	–	–	–
B.South 3–5 cm	14.85 (8.06)	97.77 (2.30)	2.23 (2.30)	0.01 (0.01)	–	–	–	–
B.West 0–3 cm	10.14 (1.07)	99.28 (0.31)	0.72 (0.31)	0.05 (0.03)	–	–	–	–
B.West 3–5 cm	9.87 (3.08)	99.43 (0.84)	0.57 (0.84)	0.00 (0.00)	–	–	–	–

MGS = median grain size, silt% = percentage silt of total, sand% = percentage sand of total, chl*a* = chlorophyll *a* concentration, TOM = wt% of total organic matter, TOC = wt% of total organic carbon, TN = wt% of total nitrogen, C:N_molar_ = molar carbon:nitrogen ratio.

### Broader geographic comparison

**Figure 6:**
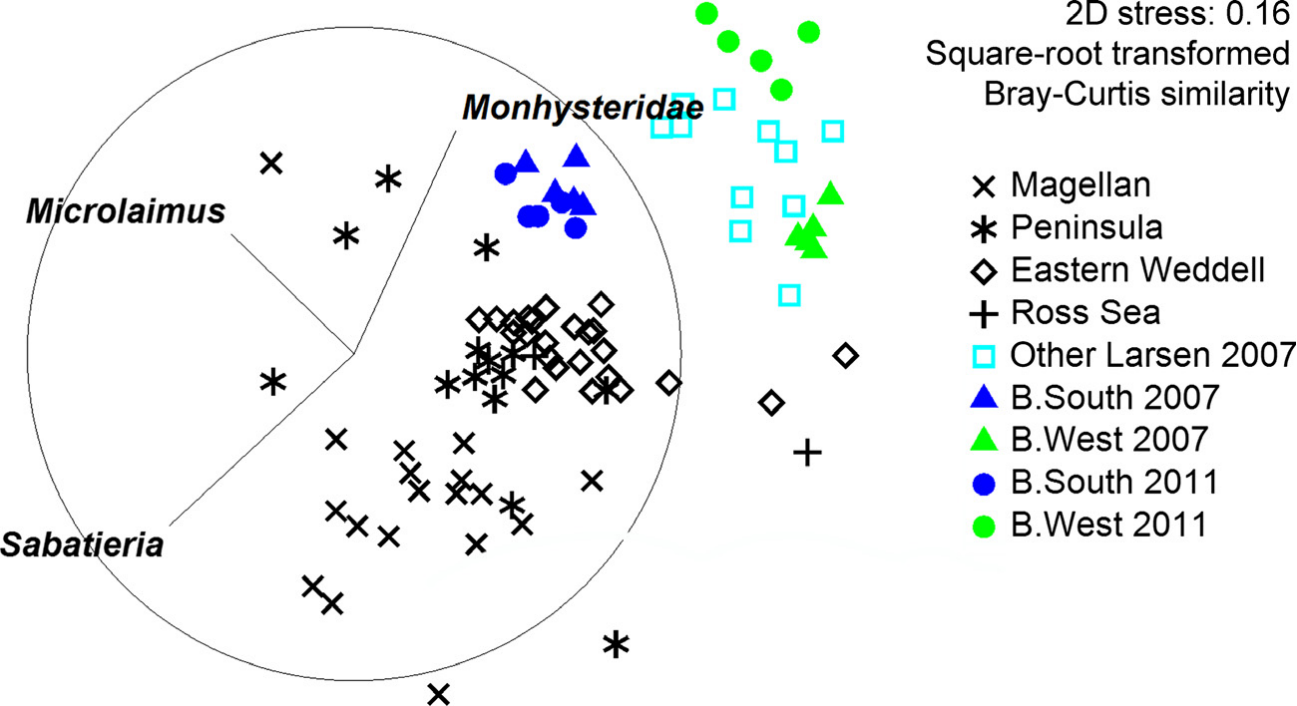
Comparison of different Antarctic shelf areas. Vector overlays represent three main contributors to community differences. Only data of 0–1000 m depth range were included in the reference database. nMDS based on Bray–Curtis similarity of square‐root transformed data.

Plotting of Larsen communities within a larger geographical context showed that, despite large dissimilarities observed within the area, communities differed substantially from those in other Antarctic shelf regions (Table 7, Fig. 6). Significant differences were found between all regions (*R* = 0.633, *P* < 0.05; one‐way ANOSIM), but they were largest between B.West and the other locations. Pairwise differences between the Larsen B stations and the other areas decreased from 2007 to 2011 for B.South, but increased for B.West (data not shown). Differences with other regions were (mostly) due to the low abundance of *Sabatieria* and high abundance of *Microlaimus* for B.South, while they were mainly attributable to high contributions of the Monhysteridae (including *Halomonhystera*) and the absence of *Sabatieria* in the case of B.West (SIMPER).

**Table 7:** Location and depth range of references included in the (sub)‐Antarctic database

Reference	Publication year	Region	Broader area	Depth range (m)	Collection year
Chen et al.	1999	Beagle Channel, Magellan Strait	Magellan	10–550 m	1994
Hauquier et al.	2011	Larsen B	Larsen 2007	~820 m	2007
Hauquier et al.	Unpublished	Drake Passage, NE Weddell Sea	Peninsula	470–520 m	2013
Ingels et al.	2006	Signy Island, South Georgia	Peninsula	~300 m	2002
Lee et al.	2001	Kapp Norvegia	Eastern Weddell	200–300 m	1998
Lee et al.	Unpublished	Bransfield Strait, Drake Passage	Peninsula	200–430 m	1998
Lee et al.	Unpublished	Kapp Norvegia, Vestkapp	Eastern Weddell	~200 m	1996
Luyten	1999	Adelaide Island	Peninsula	5–30 m	1998
Manachini	1997	Kapp Norvegia, Ross Sea	Eastern Weddell/Ross Sea	200–600 m	1994, 1996
Raes et al.	Unpublished	Elephant Island	Peninsula	~430 m	2006
Raes et al.	2010	Larsen A, Larsen B	Larsen 2007	240–430 m	2007
Vanhove et al.	1997	Kapp Norvegia, Halley Bay	Eastern Weddell	200–800 m	1989
Vanhove et al.	1998	Signy Island	Peninsula	~10 m	1994
Vanhove et al.	2004	South Sandwich Trench	Peninsula	~750 m	2002

## Discussion

Large‐scale ice‐shelf disintegration is one of the many consequences of the rapid warming trend observed along the Antarctic Peninsula. Although most of the LIS‐A/B disintegration occurred over a rather short time period (1995–2002), its effects will persist over a longer time span. Therefore, the aim of both ANT‐XXIII/8 and ANT‐XXVII/3 was to collect information at different time intervals for several components of the marine food web to be able to anticipate to future responses, and relate changes and patterns to the situation observed before (Gutt et al. 2011).

### Environmental setting and implications for benthic communities

Four years after the first sampling campaign, rapid regional warming in Antarctic Peninsula surroundings continues, evoking additional ice‐mass loss in the Larsen area (Shuman et al. 2011; Berthier et al. 2012). Consequently, seasonal phytoplankton blooms emerge (Barnes and Clarke 2011), further modifying benthic habitats at former ice‐shelf locations from an oligotrophic to a more productive state. New organic matter production in the Larsen area was demonstrated by remote sensing of net primary productivity in 1997–2011 (Cape et al. 2014), and diatom siliceous frustules found in the upper two centimeters of the sediment (i.e., the layer corresponding to post‐ice‐shelf deposition; Sañé et al. 2013). Productivity in Larsen A and B is now as high as that for other Antarctic shelf locations and tightly linked to seasonal polynya dynamics (Cape et al. 2014). Average chl*a* values reported in 2011 surface sediments (Table 6) are indeed comparable to those found elsewhere on the Antarctic shelf (e.g., Fabiano and Danovaro 1999: 0.25–0.38 *μ*g g^−1^ at 430–590 m in the Ross Sea; Vanhove et al. 2004: 0.36–0.52 *μ*g g^−1^ at 750 m in the Weddell Sea). The fivefold to tenfold increase in sediment chl*a* compared to 2007 conceivably demonstrates higher productivity in the area as more time passed since ice‐shelf collapse. However, considering a time lag between production in surface photic layers and transport of phytodetritus through the water column, summer‐bloom chlorophyll could have already reached the seafloor and benthic communities in 2011 (late‐summer sampling), but not in 2007 (early‐summer sampling). Furthermore, primary production in the Larsen area depends heavily on the sporadic breakup of seasonal sea ice, which makes food supply to the benthos hardly predictable in space and time, especially in terms of the high interannual variability (Gutt et al. 2013).

As meiofaunal assemblages are tightly linked to fresh food input (Lins et al. 2014, 2015), it is almost inevitable that the transition to a more productive (yet still highly seasonal) state will influence nematode communities (cf. TOC and chl*a* main explanatory variables in BEST results). Organic matter in surface marine sediments lies usually within the range of 0.1–5 wt%, of which the lower extreme (0.1–0.2 wt%) typically occurs in fine‐grained sediments of well‐oxygenated bathyal and abyssal depths, while average TOC values of 0.5–3 wt% dominate in deltas and on upper continental margins (Hedges and Oades 1997). Surface TOC content at B.West was thus relatively low compared to global means, while values at B.South were clearly higher, situated within the intermediate range, and comparable to values reported in other Antarctic studies at similar depths (0.2–0.75 wt%; Domack and Ishman 1997; Giordano et al. 1999).

Not only the quantity, but also the quality and source of food can influence benthic community composition. Due to the cold temperatures of Antarctic waters, phytodetritus degradation is slow, allowing its accumulation in sediment “foodbanks” (Smith et al. 2006, 2008; Mincks et al. 2008). These foodbanks can sustain a rich benthic community throughout the year (especially in long winters), even when fresh input is lacking. In addition, phytoplankton supply to the seafloor in sub‐ice zones is considerably lower than in open water owing to lower sedimentation rates (Post et al. 2007). Combining both phenomena (i.e., low degradation and sedimentation rates) and taking into account the closer connection of B.South to open water and phytodetritus input, a substantial foodbank could have developed at this site; and nematode assemblages could be feeding on organic matter that accumulated over the course of many years (cf. higher TOC and TOM; Table 6). In contrast, longer persisting ice cover at B.West prevented the establishment of an extensive foodbank, rendering communities highly dependent upon short pulses of fresh material after ice‐shelf collapse (demonstrated by higher chl*a* values).

### 2007–2011 Nematode community change

The original high dissimilarity in nematode community composition between B.South and B.West in 2007 (Raes et al. 2010) was still evident 4 years later, and temporal changes in nematode assemblages were quite different for both stations (Table 7).

Density, diversity, and generic composition at B.South remained fairly similar over the years (Figs 2 and 4; Tables 2 and 4) and changed with depth into the sediment. The community was still dominated by *Microlaimus*, an epistratum‐feeder (Wieser 1953) that is generally widespread in shallow and deep‐sea habitats (Tita et al. 2002; Gambi et al. 2003; Vanhove et al. 2004; Sebastian et al. 2007; Van Gaever et al. 2009a; Portnova et al. 2010; Vanreusel et al. 2010b). This opportunistic genus often attains elevated abundance in deeper areas that are more organically enriched (Van Gaever et al. 2004, 2006, 2009a; Sebastian et al. 2007) or recently disturbed (e.g., after iceberg scouring; Lee et al. 2001), in which case it is considered a pioneering colonizer. As there were no signs of disturbance related to iceberg scouring at the time of sampling, the first explanation seems more likely. To reach current numbers at B.South, *Microlaimus* could have benefited from lateral advective food input from the Weddell Sea during ice‐shelf cover, complemented by increased levels of phytodetritus accretion after ice‐shelf collapse. Even so, in spite of continued seasonal ice‐free periods and enhanced food conditions, changes between 2007 and 2011 nematode assemblages at B.South were not very prominent, suggesting a relatively steady community, comparable to other Antarctic shelf areas in terms of abundance and biodiversity (Raes et al. 2010). Nevertheless, generic composition at B.South was not entirely comparable to that of other Antarctic shelf areas, mainly attributable to the genera *Sabatieria* and *Microlaimus* (SIMPER; Fig. 6). Only a few individuals of *Sabatieria* were observed at B.South, while it is usually quite common in shelf samples, especially in muddy sediments (as was the case in the Larsen; Schratzberger et al. 2009; Van Gaever et al. 2009a). It tends to reside in deeper sediment layers (associated with the Redox Potential Discontinuity (RPD) layer; Vanreusel et al. 2010a; Guilini et al. 2011), where a substantial fraction of organic material becomes incorporated below the oxic zone. Perhaps, Larsen sediment conditions were not yet favorable for *Sabatieria*, as organic matter burial was rather limited in the millennia preceding ice‐shelf collision; or, alternatively, *Sabatieria* could not reach the area or establish a stable population within the 4 years time. Either way, the nematode community at B.South did not converge with other Antarctic shelf fauna as we hypothesized, although differences with other areas did decline over the years (pairwise ANOSIM).

Nematode assemblages at B.West were even more distinct from other shelf communities than at B.South (Fig. 6), as >90% of total abundance consisted of *Halomonhystera* (and *Sabatieria* was virtually absent). *Halomonhystera* is classified as a nonselective deposit feeder sensu Wieser (1953) and a general opportunistic genus (Bongers et al. 1991). Compared to the 2007 situation, densities increased drastically while diversity decreased due to proliferation of *Halomonhystera*. According to Raes et al. (2010), low density and low genus richness in 2007 reflected precollapse oligotrophic conditions. At that time, *Halomonhystera* was mainly found in deeper sediment layers (upper cm dominated by *Thalassomonhystera*), which generally contain less food. The drastic increase in *Halomonhystera* densities at station B.West over the course of only a few years is thus at least remarkable. One possible explanation is that increased direct supply of fresh food to the seabed has triggered opportunistic feeding behavior of *Halomonhystera*. Earlier research on one species of *Halomonhystera*,* H. disjuncta*, has classified it as an efficient colonizer, capable of expressing priority effects (Derycke et al. 2007b; Van Gaever et al. 2009b), a situation where first colonizing individuals have such a strong population development that they inhibit the settlement of other species. This could explain why community composition at B.West was still very different from B.South, even after a longer time period: *Microlaimus* and other genera potentially able to profit from open‐water conditions do not get a chance to settle in the *Halomonhystera*‐dominated sediments (provided that they did reach the area though; see further). Alternatively, it is possible that *Halomonhystera* is responding to sedimentary features other than fresh phytodetritus input. In fact, the subsurface (1–2 cm) maximum in *Halomonhystera* abundance strongly resembles the vertical profile observed at station Larsen B.Seep reported by Hauquier et al. (2011), where a low‐active cold seep was found (~800 m; Niemann et al. 2009). Also there, nematode assemblages were characterized by high densities, deeper density maxima, and high dominance of one *Halomonhystera* species. This prompted the question whether *Halomonhystera* depended upon chemosynthetically derived organic matter, as was the case with *Halomonhystera hermesi* (earlier identified as *H. disjuncta*) in sulphidic, microbial mat sediments at the Håkon Mosby Mud Volcano (~1300 m; Van Gaever et al. 2006). However, stable isotope data for B.Seep did not indicate such a relationship, leading to the conclusion that *Halomonhystera* thrives on phytoplanktonic rather than chemosynthetic resources (Hauquier et al. 2011). The fact that there were no signs of elevated sulfide levels, anoxia or seepage at the time of sampling at B.West further strengthens this conclusion.

Whatever the reason or mechanism behind it, the success of *Halomonhystera* at B.West in 2011 further isolated the community from B.South (and by extension any other Antarctic shelf region) compared to 2007. Instead of anticipated convergence of communities at both stations, they increasingly diverged from each other.

### Nematode colonization dynamics

Besides food availability as a local, environmental driver for differences between areas, also more regional processes such as colonization ability of organisms can structure benthic communities. Marine nematode dispersal is dependent on body morphology, swimming ability, and feeding strategies (Thomas and Lana 2011), and as nematodes lack pelagic larvae or propagules, dispersal is in this case synonymous to gene flow (Derycke et al. 2013). It was already shown that nematode colonization is a slow process (Post et al. 2007), predominantly driven by passive transport via bottom currents (Boeckner et al. 2009); and not necessarily related to higher food input (e.g., Guilini et al. 2011). Furthermore, colonization dynamics depend on the distance (Derycke et al. 2007a), proximity of a source population, and the time needed for successful settlement and reproduction (Schratzberger et al. 2006; Raes et al. 2010). Closer connection of B.South to the open Weddell Sea as a source for new recruits may therefore partly explain observed differences with B.West. Raes et al. (2010) calculated a speed of recovery of 60.8 m year^−1^, and hence, approximately 1000 years needed to cross the distance of 70.8 km between B.West and B.South. So far, too little time has passed for the nematodes to travel between both Larsen stations on one hand and between larger geographical areas on the other hand. With time, and if local conditions allow it, maybe *Microlaimus* will be able to reach the innermost part of the Larsen B, and maybe other genera, such as *Sabatieria*, will find their way into Larsen.

### Comparison with other benthic groups

Nematodes are only one taxonomic player in the Antarctic marine benthic food web, and it can be valuable to compare their response with other food‐web compartments, as changes at one trophic level may impact other faunal components (either bottom‐up or top‐down) or remineralization processes in the sediment (e.g., Moline et al. 2004; Montes‐Hugo et al. 2009). As already shown in 2007 (Gutt et al. 2011), different benthic components react in different ways to the ice‐shelf collapse, each at their own pace (some organisms are more sensitive to disturbance, especially long‐lived taxa such as Porifera). Results on other trophic levels for the 2011 expedition remain scarce so far, but Gutt et al. (2013) found a drastic decrease in the aggregations of two ascidians between 2007 and 2011 but an increase in abundances of deposit‐feeding ophiuroids. Although they could not relate their findings to particular environmental characteristics, it clearly shows the high dynamics of Antarctic benthos and the probability for both negative and positive effects to arise after large‐scale alterations. Together with this study, their research highlights the difficulties to relate changes in faunal communities to environmental factors because benthic responses may take a long time and are highly variable.

## Conflict of Interest

None declared.

## References

[ece31869-bib-0001] Anderson, M. , R. Gorley , and K. Clarke . 2008 PERMANOVA for PRIMER, guide to software and statistical methods. PRIMER‐E Ltd, Plymouth 214 p.

[ece31869-bib-0002] Barnes, D. K. , and A. Clarke . 2011 Antarctic marine biology. Curr. Biol. 21:R451–R457. 21683895 10.1016/j.cub.2011.04.012

[ece31869-bib-0003] Barnett, P. R. O. , J. Watson , and D. Connelly . 1984 A multiple corer for taking virtually undisturbed samples from shelf, bathyal and abyssal sediments. Oceanol. Acta 7:399–408.

[ece31869-bib-0004] Berthier, E. , T. Scambos , and C. Shuman . 2012 Mass loss of Larsen B tributary glaciers (Antarctic Peninsula) unabated since 2002. Geophys. Res. Lett. 39:1–18.

[ece31869-bib-0005] Bertolin, M. L. , and I. R. Schloss . 2009 Phytoplankton production after the collapse of the Larsen A Ice Shelf, Antarctica. Polar Biol. 32:1435–1446.

[ece31869-bib-0006] Boeckner, J. B. , J. Sharma , and H. C. Proctor . 2009 Revisiting the meiofauna paradox, dispersal and colonisation of nematodes and other meiofaunal organisms in low‐ and high‐energy environments. Hydrobiologia 624:91–106.

[ece31869-bib-0007] Bongers, T. , R. Alkemade , and G. W. Yeates . 1991 Interpretation of disturbance‐induced maturity decrease in marine nematode assemblages by means of the Maturity Index. Mar. Ecol. Prog. Ser. 76:135–142.

[ece31869-bib-0008] Cape, M. R. , M. Vernet , M. Kahru , and G. Spreen . 2014 Polynya dynamics drive primary production in the Larsen A and B embayments following ice shelf collapse. J. Geophys. Res. Oceans 119:572–594.

[ece31869-bib-0009] Chen, G. T. , R. L. Herman , and M. Vincx . 1999 Meiofauna communities from the Straits of Magellan and the Beagle Channel. Sci. Mar. 63:123–132.

[ece31869-bib-0010] Clarke, K. R. , and R. N. Gorley (2006). PRIMER v6, User manual/tutorial. PRIMER‐E, Plymouth, UK 190 p.

[ece31869-bib-0011] Clarke, A. , E. J. Murphy , M. P. Meredith , J. C. King , L. S. Peck , D. K. A. Barnes , et al. 2007 Climate change and the marine ecosystem of the western Antarctic Peninsula. Philos. Trans. R. Soc. B 362:149–166. 10.1098/rstb.2006.1958 PMC1764833 17405211

[ece31869-bib-0012] Derycke, S. , R. Van Vynckt , J. Vanoverbeke , M. Vincx , and T. Moens . 2007a Colonization patterns of Nematoda on decomposing algae in the estuarine environment, community assembly and genetic structure of the dominant species *Pellioditis marina* . Limnol. Oceanogr. 52:992–1001.

[ece31869-bib-0013] Derycke, S. , T. Backeljau , C. Vlaeminck , A. Vierstraete , J. Vanfleteren , M. Vincx , et al. 2007b Spatiotemporal analysis of population genetic structure in *Geomonhystera disjuncta* (Nematoda, Monhysteridae) reveals high levels of molecular diversity. Mar. Biol. 151:1799–1812.

[ece31869-bib-0014] Derycke, S. , T. Backeljau , and T. Moens . 2013 Dispersal and gene flow in free‐living marine nematodes. Front. Zool. 10:1 http://www.frontiersinzoology.com/content/10/1/1. 23356547 10.1186/1742-9994-10-1 PMC3567977

[ece31869-bib-0015] Domack, E. , and S. Ishman . 1997 Oceanographic and physiographic controls on modern sedimentation within Antarctic fjords. Geol. Soc. Am. Bull. 105:1175–1189.

[ece31869-bib-0016] Domack, E. , D. Duran , A. Leventer , S. Ishman , S. Doane , S. McCallum , et al. 2005 Stability of the Larsen B ice shelf on the Antarctic Peninsula during the Holocene epoch. Nature 436:681–685. 16079842 10.1038/nature03908

[ece31869-bib-0017] Fabiano, M. , and R. Danovaro . 1999 Meiofauna distribution and mesoscale variability in two sites of the Ross Sea (Antarctica) with contrasting food supply. Polar Biol. 22:115–123.

[ece31869-bib-0018] Gambi, C. , A. Vanreusel , and R. Danovaro . 2003 Biodiversity of nematode assemblages from deep‐sea sediments of the Atacama Slope and Trench (South Pacific Ocean). Deep‐Sea Res. I 50:103–117.

[ece31869-bib-0019] Gilbert, R. , and E. W. Domack . 2003 Sedimentary record of disintegrating ice shelves in a warming climate, Antarctic Peninsula. Geochem. Geophys. Geosyst. 4:1038–1050.

[ece31869-bib-0020] Giordano, R. , G. Lombardi , L. Ciaralli , E. Beccaloni , A. Sepe , M. Ciprotti , et al. 1999 Major and trace elements in sediments from Terra Nova Bay, Antarctica. Sci. Total Environ. 227:29–40.

[ece31869-bib-0021] Guilini, K. , T. Soltwedel , D. Van Oevelen , and A. Vanreusel . 2011 Deep‐sea nematodes actively colonise sediments, irrespective of the presence of a pulse of organic matter, results from an in‐situ experiment. PLoS ONE 6:e18912. 21526147 10.1371/journal.pone.0018912 PMC3079745

[ece31869-bib-0022] Gutt, J. , A. Starmans , and G. Dieckmann . 1996 Impact of iceberg scouring on polar benthic habitats. Mar. Ecol. Prog. Ser. 137:311–316.

[ece31869-bib-0023] Gutt, J. , I. Barratt , E. Domack , C. d'Udekem d'Acoz , W. Dimmler , A. Grémare , et al. 2011 Biodiversity change after climate‐induced ice‐shelf collapse in the Antarctic. Deep‐Sea Res. II 58:74–83.

[ece31869-bib-0024] Gutt, J. , M. Cape , W. Dimmler , L. Fillinger , E. Isla , V. Lieb , et al. 2013 Shifts in Antarctic megabenthic structure after ice‐shelf disintegration in the Larsen area east of the Antarctic Peninsula. Polar Biol. 36:895–906.

[ece31869-bib-0025] Hauquier, F. , J. Ingels , J. Gutt , M. Raes , and A. Vanreusel . 2011 Characterisation of the nematode community of a low‐activity cold seep in the recently ice‐shelf free Larsen B area, Eastern Antarctic Peninsula. PLoS ONE 6:e22240. 21799799 10.1371/journal.pone.0022240 PMC3140504

[ece31869-bib-0026] Hedges, J. I. , and J. M. Oades . 1997 Comparative organic geochemistries of soils and marine sediments. Org. Geochem. 27:319–361.

[ece31869-bib-0027] Heip, C. , M. Vincx , and G. Vranken . 1985 The ecology of marine nematodes. Oceanogr. Marine Biol. 23:399–489.

[ece31869-bib-0028] Higgins, R. P. , and H. Thiel . 1988 Introduction to the study of Meiofauna. Smithsonian Institution Press, London 488 p.

[ece31869-bib-0029] Ingels, J. , S. Vanhove , I. De Mesel , and A. Vanreusel . 2006 The biodiversity and biogeography of the free‐living nematode genera Desmodora and Desmodorella (family Desmodoridae) at both sides of the Scotia Arc. Polar Biol. 29:936–949.

[ece31869-bib-0030] Lee, H. J. , S. Vanhove , L. S. Peck , and M. Vincx . 2001 Recolonisation of meiofauna after catastrophic iceberg scouring in shallow Antarctic sediments. Polar Biol. 24:918–925.

[ece31869-bib-0031] Lins, L. , K. Guilini , G. Veit‐Köhler , F. Hauquier , R. M. S. Alves , A. M. Esteves , et al. 2014 The link between meiofauna and surface productivity in the Southern Ocean. Deep‐Sea Res. II 108:60–68.

[ece31869-bib-0032] Lins, L. , M. C. da Silva , F. Hauquier , A. M. Esteves , and A. Vanreusel . 2015 Nematode community composition and feeding shaped by contrasting productivity regimes in the Southern Ocean. Prog. Oceanogr. 134:356–369.

[ece31869-bib-0033] Luyten (1999) Meiofauna van Antarctica, structurele en trofische aspecten. MSc. Thesis, Ghent University, 89 p.

[ece31869-bib-0034] Manachini (1997) Biodiversity of Nematoda assemblages in the Antarctic sea bed. MSc. Thesis, Ghent University, 72 p.

[ece31869-bib-0035] Mincks, S. L. , C. R. Smith , R. M. Jeffreys , and P. Y. G. Sumida . 2008 Trophic structure on the West Antarctic Peninsula shelf, Detritivory and benthic inertia revealed by d13C and d15N analysis. Deep‐Sea Res. II 55:2502–2514.

[ece31869-bib-0036] Moline, M. A. , H. Claustre , T. K. Frazer , O. Schofield , and M. Vernet . 2004 Alteration of the food web along the Antarctic Peninsula in response to a regional warming trend. Glob. Change Biol. 10:1973–1980.

[ece31869-bib-0037] Montes‐Hugo, M. , S. C. Doney , H. W. Ducklow , W. Fraser , D. Martinson , S. E. Stammerjohn , et al. 2009 Recent changes in phytoplankton communities associated with rapid regional climate change along the western Antarctic Peninsula. Science 323:1470–1473. 19286554 10.1126/science.1164533

[ece31869-bib-0038] Niemann, H. , D. Fischer , D. Graffe , K. Knittel , A. Montiel , O. Heilmayer , et al. 2009 Biogeochemistry of a low‐activity cold seep in the Larsen B area, western Weddell Sea, Antarctica. Biogeosciences 6:2383–2395.

[ece31869-bib-0039] Portnova, D. , V. Mokievsky , and T. Soltwedel . 2010 Nematode species distribution patterns at the Håkon Mosby Mud Volcano (Norwegian Sea). Mar. Ecol. 2010:1–18.

[ece31869-bib-0040] Post, A. L. , M. A. Hemer , P. E. O'Brien , D. Roberts , and M. Craven . 2007 History of benthic colonization beneath the Amery ice shelf, East Antarctica. Mar. Ecol. Prog. Ser. 344:29–37.

[ece31869-bib-0041] R Core Team (2013) R: a language and environment for statistical computing. R Foundation for Statistical Computing, Vienna, Austria URL http://www.R-project.org/.

[ece31869-bib-0042] Rack, W. , and H. Rott . 2004 Pattern of retreat and disintegration of the Larsen B ice shelf. Antarctic Peninsula. Ann. Glaciol. 39:505–510.

[ece31869-bib-0043] Raes, M. , A. Rose , and A. Vanreusel . 2010 Response of nematode communities after large‐scale ice‐shelf collapse events in the Antarctic Larsen area. Glob. Change Biol. 16(5):1618–1631.

[ece31869-bib-0044] Rebesco, M. , E. Domack , F. Zgur , C. Lavoie , A. Leventer , S. Brachfeld , et al. 2014 Boundary condition of grounding lines prior to collapse, Larsen‐B Ice Shelf, Antarctica. Science 345:1354–1358. 25214629 10.1126/science.1256697

[ece31869-bib-0045] Rott, H. , F. Müller , T. Nagler , and D. Floricioiu . 2011 The imbalance of glaciers after disintegration of Larsen B ice shelf, Antarctic Peninsula. Cryosphere 5:125–134.

[ece31869-bib-0046] Sañé, E. , E. Isla , M. Angeles‐Bárcena , and D. J. DeMaster . 2013 A shift in the biogenic silica of sediment in the Larsen B continental shelf, off the Eastern Antarctic Peninsula, resulting from climate change. PLoS ONE 8:e52632. 23300983 10.1371/journal.pone.0052632 PMC3534681

[ece31869-bib-0047] Scambos, T. A. , J. A. Bohlander , C. A. Shuman , and P. Skvarca . 2004 Glacier acceleration and thinning after ice shelf collapse in the Larsen B embayment, Antarctica. Geophys. Res. Lett. 31:L18402.

[ece31869-bib-0048] Schratzberger, M. , K. Warr , and S. I. Rogers . 2006 Patterns of nematode populations in the southwestern North Sea and their link to other components of the benthic fauna. J. Sea Res. 55(2):113–127.

[ece31869-bib-0049] Schratzberger, M. , N. Lampadariou , P. J. Somerfield , L. Vandepitte , and E. Vanden Berghe . 2009 The impact of seabed disturbance on nematode communities, linking field and laboratory observations. Mar. Biol. 156:709–724.

[ece31869-bib-0050] Sebastian, S. , M. Raes , I. De Mesel , and A. Vanreusel . 2007 Comparison of the nematode fauna from the Weddell Sea Abyssal Plain with two North Atlantic abyssal sites. Deep‐Sea Res. II 54:1727–1736.

[ece31869-bib-0051] Seinhorst, J. W. 1959 A rapid method for the transfer of nematodes from fixative to anhydrous glycerine. Nematologica 4:67–69.

[ece31869-bib-0052] Shuman, C. A. , E. Berthier , and T. A. Scambos . 2011 2001–2009 elevation and mass losses in the Larsen A and B embayments, Antarctic Peninsula. J. Glaciol. 57:737–754.

[ece31869-bib-0053] Smith, C. R. , S. Mincks , and D. J. DeMaster . 2006 A synthesis of bentho‐pelagic coupling on the Antarctic shelf, Food banks, ecosystem inertia and global climate change. Deep‐Sea Res. II 53:875–894.

[ece31869-bib-0054] Smith, C. R. , S. Mincks , and D. J. DeMaster . 2008 The FOODBANCS project, Introduction and sinking fluxes of organic carbon, chlorophyll‐a and phytodetritus on the western Antarctic Peninsula continental shelf. Deep‐Sea Res. II 55:2404–2414.

[ece31869-bib-0055] Thomas, M. C. , and P. C. Lana . 2011 A new look into the small‐scale dispersal of free‐living marine nematodes. Zoologia 28:449–456.

[ece31869-bib-0056] Tita, G. , G. Desrosiers , M. Vincx , and M. Clément . 2002 Intertidal meiofauna of the St Lawrence estuary (Quebec, Canada), diversity, biomass and feeding structure of nematode assemblages. J. Mar. Biol. Assoc. U.K. 82:779–791.

[ece31869-bib-0057] Van Gaever, S. , A. Vanreusel , J. A. Hughes , B. J. Bett , and K. Kiriakoulakis . 2004 The macro‐ and micro‐scale patchiness of meiobenthos associated with the Darwin Mounds (north‐east Atlantic). J. Mar. Biol. Assoc. U.K. 84:547–556.

[ece31869-bib-0058] Van Gaever, S. , L. Moodley , D. de Beer , and A. Vanreusel . 2006 Meiobenthos at the Arctic Håkon Mosby Mud Volcano, with a parental‐caring nematode thriving in sulphide‐rich sediments. Mar. Ecol. Prog. Ser. 321:143–155.

[ece31869-bib-0059] Van Gaever, S. , J. Galéron , M. Sibuet , and A. Vanreusel . 2009a Deep‐sea habitat heterogeneity influence on meiofaunal communities in the Gulf of Guinea. Deep‐Sea Res. II 56:2259–2269.

[ece31869-bib-0060] Van Gaever, S. , K. Olu , S. Derycke , and A. Vanreusel . 2009b Metazoan meiofaunal communities at cold seeps along the Norwegian margin, influence of habitat heterogeneity and evidence for connection with shallow‐water habitats. Deep‐Sea Res. I 56:772–785.

[ece31869-bib-0061] Vanaverbeke, J. , T. N. Bezerra , U. Braeckman , A. De Groote , N. De Meester , T. Deprez , et al. (2014) NeMys, World Database of Free‐Living Marine Nematodes. Available at: http://www.nemys.ugent.be (Accessed 24 September 2014).

[ece31869-bib-0062] Vanhove, S. , J. Wittoeck , M. Beghyn , D. Van Gansbeke , A. Van Kenhove , A. Coomans , et al. 1997 Role of the meiobenthos in Antarctic ecosystems Pp. 326–386. *in* Caschetto S., ed. Belgian research programme on the Antarctic, scientific results of phase III (1992‐1996), 1. Marine biochemistry and ecodynamics. University of Ghent, Belgium. (1997). A3/02/001/1‐59.

[ece31869-bib-0063] Vanhove, S. , H. J. Lee , M. Beghyn , D. Van Gansbeke , S. Brockington , and M. Vincx . 1998 The metazoan meiofauna in its biogeochemical environment, the case of an Antarctic coastal sediment. J. Mar. Biol. Assoc. U.K. 78:411–434.

[ece31869-bib-0064] Vanhove, S. , H. Vermeeren , and A. Vanreusel . 2004 Meiofauna towards the South Sandwich Trench (750‐6300 m), focus on nematodes. Deep‐Sea Res. II 51:1665–1687.

[ece31869-bib-0065] Vanreusel, A. , A. De Groote , S. Gollner , and M. Bright . 2010a Ecology and biogeography of free‐living nematodes associated with chemosynthetic environments in the deep sea: a review. PLoS ONE 5:e12449. 20805986 10.1371/journal.pone.0012449 PMC2929199

[ece31869-bib-0066] Vanreusel, A. , G. Fonseca , R. Danovaro , et al. 2010b The contribution of deep‐sea macrohabitat heterogeneity to global nematode diversity. Mar. Ecol. 31:6–20.

[ece31869-bib-0067] Vaughan, D. G. , G. J. Marshall , W. M. Connolley , C. Parkinson , R. Mulvaney , D. A. Hodgson , et al. 2003 Recent rapid regional climate warming on the Antarctic Peninsula. Clim. Change. 60:243–274.

[ece31869-bib-0068] Vincx, M. 1996 Meiofauna in marine and freshwater sediments Pp. 187–195. *in* Hall G. S., ed. Methods for the examination of organismal diversity in soils and sediments. Cab International, University Press, Cambridge.

[ece31869-bib-0069] Warwick, R. M. , H. M. Platt , and P. J. Somerfield . 1998 Free‐living marine nematodes part III Monhysterids: pictorial key to world genera and notes for the identification of British species Synopses of the British fauna (new series) 53. Field Studies Council, Shrewsbury, UK, 296 p.

[ece31869-bib-0070] Wentworth, C. K. 1922 A scale of grade and class terms for clastic sediments. J. Geol. 30:377–392.

[ece31869-bib-0071] Wieser, W. 1953 Die Beziehung zwischen Mundhöhlengestalt, Ernährungsweise und Vorkommen bei freilebenden marinen Nematoden. Arkiv för Zoologi 4:439–484.

